# Head-to-Head Comparison of [^68^ Ga]Ga-FAPI-46-PET/CT and [^18^F]F-FDG-PET/CT for Radiotherapy Planning in Head and Neck Cancer

**DOI:** 10.1007/s11307-022-01749-7

**Published:** 2022-06-30

**Authors:** Simone Wegen, Lutz van Heek, Philipp Linde, Karina Claus, Dennis Akuamoa-Boateng, Christian Baues, Shachi Jenny Sharma, Klaus Schomäcker, Thomas Fischer, Katrin Sabine Roth, Jens Peter Klußmann, Simone Marnitz, Alexander Drzezga, Carsten Kobe

**Affiliations:** 1 Department of Radiation Oncology, Cyberknife and Radiotherapy, University Hospital Cologne, Kerpener Str. 62, 50937 Cologne, Germany; 2 Department of Nuclear Medicine, Faculty of Medicine and University Hospital Cologne, University of Cologne, Cologne, Germany; 3 Department of Otolaryngology-Head and Neck Surgery, University Hospital of Cologne, Cologne, Germany; 4 Center for Molecular Medicine, Medical Faculty, University Hospital Cologne, Cologne, Germany; 5 German Center for Neurodegenerative Diseases (DZNE), Bonn-Cologne, Germany; 6 Institute of Neuroscience and Medicine, Molecular Organization of the Brain, Forschungszentrum Jülich, INM-2), Cologne, Germany

**Keywords:** FDG/PET, Head and neck cancer, FAPI, Radiotherapy planning, PET-based

## Abstract

**Introduction:**

In head and neck cancers (HNCs), fibroblast activation protein (FAP) is expressed by cancer-associated fibroblasts (CAFs) in the tumor microenvironment. Preliminary evidence suggests that detection and staging is feasible with positron emission tomography (PET/CT) imaging using [^68^ Ga]-radiolabeled inhibitors of FAP ([^68^ Ga]Ga-FAPI-46) in HNCs. This study aims to compare [^68^ Ga]Ga-FAPI-46 PET/CT and [^18^F]-fluorodeoxy-d-glucose ([^18^F]F-FDG) PET/CT with a focus on improved target volume definition and radiotherapy planning in patients with HNC referred for chemoradiation.

**Methods:**

A total of 15 patients with HNCs received both [^68^ Ga]Ga-FAPI-46 PET/CT and [^18^F]F-FDG PET/CT with a thermoplastic mask, in addition to initial tumor staging by conventional imaging with contrast-enhanced CT and/or MRI. Mean intervals between FAPI/FDG and FAPI/conventional imaging were 4 ± 20 and 17 ± 18 days, respectively. Location and number of suspicious lesions revealed by the different procedures were recorded. Subsequently, expert-generated gross tumor volumes (GTVs) based on conventional imaging were compared to those based on [^18^F]F-FDG and [^68^ Ga]Ga-FAPI-46 PET/CT to measure the impact on subsequent radiation planning.

**Results:**

All patients had focal FAPI uptake above background in tumor lesions. Compared to FDG, tumor uptake (median SUVmax 10.2 vs. 7.3, *p* = 0.008) and tumor-to-background ratios were significantly higher with FAPI than with FDG (SUVmean liver: 9.3 vs. 3.2, *p* < 0.001; SUVmean bloodpool: 6.9 vs. 4.0, *p* < 0.001). A total of 49 lesions were recorded. Of these, 40 (82%) were FDG^+^ and 41 (84%) were FAP^+^. There were 5 (10%) FAP^+^/FDG^−^ lesions and 4 (8%) FAP^−^/FDG^+^ lesions. Volumetrically, a significant difference was found between the GTVs (median 57.9 ml in the FAPI-GTV, 42.5 ml in the FDG-GTV, compared to 39.2 ml in the conventional-GTV). Disease stage identified by FAPI PET/CT was mostly concordant with FDG PET/CT. Compared to conventional imaging, five patients (33%) were upstaged following imaging with FAPI and FDG PET/CT.

**Conclusion:**

We demonstrate that [^68^ Ga]Ga-FAPI-46 -PET/CT is useful for detecting tumor lesions in patients with HNCs. There is now a need for prospective randomized studies to confirm the role of [^68^ Ga]Ga-FAPI-46 PET/CT in relation to [^18^F]F-FDG PET/CT in HNCs and to evaluate its impact on clinical outcome.

## Introduction 

Head and neck cancers (HNCs) rank sixth in the most common cancer entities and account for over 300,000 deaths per year worldwide [1]. Over 90% of all HNCs are squamous cell carcinomas (SCCs) with poor prognosis at advanced tumor stages [2]. Accurate staging is particularly important if patients are to be offered the right treatment: operative resection of the tumor region and affected lymph nodes at early tumor stages (cT1-3, cN0-2, cM0) can be considered based on the patient’s general condition and tumor site. Definitive chemoradiotherapy is a viable option for more advanced tumor stages (≥ cT3, ≥ cN2, cM0). However, modified strategies can also be considered, depending on patient preference (especially when function-maintaining operation is not possible), previous illnesses, expected complications during anesthesia, or other factors.

In HNCs, [^18^F]F-FDG PET/CT not only serves for staging purposes but also benefits target volume definition and is well established in (radio-)oncologic management: a substantial body of evidence available shows how target volume definition using MRI and [^18^F]F-FDG PET/CT can improve local control and disease-free survival [3–5]. However, the efficacy of FDG PET/CT can be reduced in HNCs through tracer background uptake in salivary glands, brain and sometimes soft tissue, muscles, or even lymph nodes [6], the rates of false-negative and false-positive findings with FDG PET/CT being estimated at around 6% and 20%, respectively [7].

Fibroblast-activation-protein (FAP) is overexpressed by cancer-associated fibroblasts (CAFs) in the tumor microenvironment [8]. PET/CT using [^68^ Ga]Ga-labeled inhibitors of FAP ([^68^ Ga]Ga-FAPI-46) visualizes CAFs. Through this, [^68^ Ga]Ga-FAPI-46 PET/CT is able to detect several tumor entities such as HNCs with a high tumor-to-background contrast. [^68^ Ga]Ga-FAPI-46-has proven particularly suitable as a tracer here [9, 10]. Previous studies have indicated that [^18^F]F-FDG PET/CT allows highly specific imaging of HNCs, and it is utilized in various clinical scenarios [11]. Here, uptake of FAPI ligands in HNCs, as reported by the Heidelberg group, has the potential to improve the detection rate of nodal metastasis and optimize radiotherapy planning [12].

In this study, we aim to provide an intra-individual comparison of [^68^ Ga]Ga-FAPI-46 PET/CT and [^18^F]F-FDG PET/CT and assess the impact of the former on target volume definition and tumor stage.

## Methods

We retrospectively reviewed our clinical database for patients who had received additional [^68^ Ga]Ga-FAPI-46 PET/CT to complement radiotherapeutic planning between March and November 2020. All patients received planning-CTs with contrast agent and a pretherapeutic MRI. We performed [^18^F]F-FDG and [^68^ Ga]Ga-FAPI-46 PET/CT scans for treatment planning. The scheduling of the two scanning procedures was kept as close as possible (median time between scans: 4 days, range 2–59 days). Two scans were never performed on the same day. All clinical investigations were conducted according to Declaration of Helsinki principles. All procedures were performed in compliance with the regulations of the responsible local authorities, and the local institutional review board waived the requirement for additional approval owing to the retrospective character of this study. All patients gave written informed consent to PET imaging and inclusion of their data in retrospective scientific analyses.

### PET Imaging and Biodistribution Analysis

All PET/CT examinations were performed on a Biograph mCT Flow–Edge 128 PET/CT system (Siemens Medical Solutions) with a 128-slice spiral CT component from the base of the skull to the mid-thigh or skull-vertex to the mid-thigh, depending on the suspected tumor site. The CT scan for attenuation correction was performed as a native non-diagnostic scan with a tube current of 30 mAs and a maximum voltage power of 120 kVp. This CT scan was followed by a PET emission scan. To meet the criteria for European Association of Nuclear Medicine and its Research Ltd. (EARL) certification, reconstruction for quantitative analyses was performed via ordered subset expectation maximization (OSEM) algorithm (four iterations and twelve subsets), followed by an intrinsic 5-mm Gaussian filter in all directions.

[^68^ Ga]Ga-FAPI-46 PET/CT scans were performed following mean i.v. administration of 147 MBq [^68^ Ga]Ga-FAPI-46. On average, 263 MBq [^18^F]F-FDG were used for [^18^F]F-FDG PET/CT scans.

Images were interpreted by experienced nuclear medicine physicians (L.v.H. and C.K.). For lesion-based analysis, lesions were labeled as positive or negative by [^18^F]F-FDG PET/CT (FDG^+^ or FDG^−^) and also as positive or negative by [^68^ Ga]Ga-FAPI-46 PET/CT (FAP^+^ or FAP^−^). For quantitative evaluation, the maximal standardized uptake value (SUVmax) was measured in all tumor lesions. The mean standardized uptake value (SUVmean) in the reference regions was determined by placing a sphere of 3 cm diameter in the upper right part of the liver and in the mediastinum (refers to ascending aorta).

### Target Volume Delineation

Varian medical systems (Siemens Healthineers) treatment planning system (TPS) ‘Eclipse’ was used for target volume delineation and matching of the available imaging. By ensuring that the positioning for scanning of all patients was exactly the same as that used for their subsequent radiotherapy (using a long thermoplastic mask), we were able to achieve exact matching of the images. First, a straight contrast-CT and MRI gross tumor volume (GTV) (conv-GTV) was created. Secondly, an [^18^F]F-FDG PET/CT-based GTV was drawn (FDG-GTV), and thirdly, an experimental [^68^ Ga]Ga-FAPI-46 PET/CT based GTV was created (FAPI-GTV). A window of SUVmax 0–5 was employed for PET/CT-based contouring. All GTVs were defined in accordance with the latest EORTC contouring guidelines for HNCs, and each structure set was approved by a board-certified radiation oncologist (S.K.) [13]. Clinical target volume (CTV) was delineated based on anatomical expansion of the GTV (+ 5 mm) using compartmentalization of head and neck anatomy. As in clinical practice, the immobilization device enabled us to achieve a CTV-to-PTV-margin of 4 mm.

To avoid upstaging of equivocal cases, [^68^ Ga]Ga-FAP-46-positivity was carefully considered for final contouring, especially in the case of only low-to-moderate positive [^18^F]F-FDG PET findings. Experienced physicians, board-certified for nuclear medicine and radiation oncology, evaluated the two images independently for each patient. Interpretation of discordant lesions (FAP + but FDG −) took into consideration potential pitfalls, i.e., scar tissue or other benign causes of FAP-uptake. Most importantly, lesions were correlated with CT imaging to confirm FDG or FAP-positive findings.

No upstaging or treatment decisions were based on [^68^ Ga]Ga-FAPI-46 imaging information alone but took into consideration the results of clinical examination and CT and MRI imaging and were discussed in multidisciplinary meetings with an otolaryngologist, physicians for nuclear medicine, and radiation oncologists in consensus. Where FAP-imaging was the only modality suggesting nodal upstaging or new metastatic disease and where biopsy evidence was lacking, we decided not to treat a patient according to the FAP results.

### Statistics

Descriptive statistics were used to present patient characteristics and results. Wilcoxon matched-pairs signed-rank tests were used to check for significant differences between continuous variables. Spearman’s rank correlation was used to correlate GTVs. A *p*-value smaller than 0.05 was regarded as statistically significant. All statistical analyses were performed using R-Statistics.

## Results 

Fifteen patients were available for this study (twelve men and three women; see Table 1) who had received both [^18^F]F-FDG PET/CT and [^68^ Ga]Ga-FAPI-46 PET/CT between March and November 2020. All patients had histologically confirmed HNCs prior to imaging and had been referred by the local multidisciplinary board for radio(chemo-/immuno)therapy, in most cases (14 out of 15 cases) because they had reached inoperable stages of disease (UICC stages III and IV). One patient had a resectable tumor (laryngeal carcinoma, cT2 cN0) but preferred radiochemotherapy over operation for better functional preservation of the voice. Most patients (93.3%) had a histopathological diagnosis of SCC, and 33.3% were HPV-positive.

**Table 1:** Patient characteristics

	Overall (*n* = 15)
**Age**	
Median [Min, Max]	66.0 [37.0, 82.0]
**Sex**	
Male	12 (80.0%)
Female	3 (20.0%)
**BMI**	
Mean (SD)	25.2 (6.61)
Median [Min, Max]	23.3 [14.8, 40.2]
**Primary tumor site**	
Nasopharynx	3 (20.0%)
Oropharynx	8 (53.3%)
Hypopharynx	1 (6.7%)
Larynx	3 (20.0%)
**Histology**	
Adeno	1 (6.7%)
Squamous	14 (93.3%)
**HPV status**	
Negative	7 (46.7%)
Positive	5 (33.3%)
Not reported	3 (20.0%)
**Treatment**	
Chemoradiotherapy (CRT)	9 (60.0%)
Radiotherapy & Antibody therapy	4 (26.7%)
Radiotherapy (RT)	2 (13.3%)

*BMI* body mass index, *HPV* human papilloma virus

### Biodistribution

For [^68^ Ga]Ga-FAPI-46, the highest uptake was observed in the primary tumor region (*n* = 15, median SUVmax 14.8, range: 9.26–26.6) with the second highest in the nodal metastasis (*n* = 23) (median SUVmax 9.47, range 1.83–24.9). Uptake in visceral metastasis (*n* = 7) was 7.05 (median SUV max, range: 1.8–25.0) while in bone metastasis (*n* = 3), it was 7.45 (median SUVmax, range: 4.0–14.2). Background uptake was assessed in liver (median SUVmean 1.13, range: 0.4–3.03) and in the bloodpool (median SUVmean 1.49, range: 0.97–2.56).

For [^18^F]F-FDG, the highest uptake was observed in the primary tumor (median SUVmax 13.4, range: 5.68–21.9) and next highest in lymph node metastasis (*n* = 23) (median SUVmax 6.17, range 1.73–20.9).

The median SUVmax in visceral metastasis (*n* = 7) was 5.57 (range: 2.62–11.1) while in bone metastasis (*n* = 3), it was 2.59 (median SUVmax, range: 1.41–2.75).

**Fig. 1:**
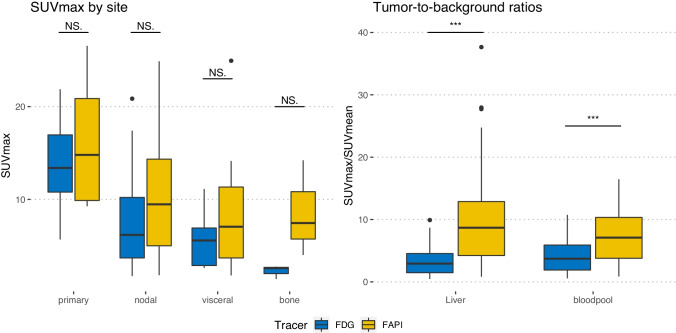
Uptake measurements in 49 lesions. Left part: SUVmax by site for FDG (blue box) and FAPI (yellow box) in tumorous lesions. Right part: Tumor-to-background (TBR) ratios (SUVmax/SUVmean) for FDG (median 2.94; blue box) and FAPI (median 8.70; yellow box; *p* < 0.001). The statistical comparison was performed by a Wilcoxon paired rank test. NS. not significant; ***p < 0.001.

Background uptake in the liver was 2.58 (median SUVmax, range 1.35–3.88) on FDG and 1.13 (median SUVmax, range 0.4–3.03) on [^68^ Ga]Ga-FAPI-46 PET/CT. The uptake in the mediastinal bloodpool was 2.12 (median SUVmax, range 1.26–2.88) for FDG and 1.49 (median SUVmax, range 0.97–2.56) for FAPI. Table 2 shows biodistribution measurements for [^68^ Ga]Ga-FAPI-46 PET/CT and FDG PET/CT. Tumor-to-background ratios are presented in Table 3 and Fig. 1.

**Table 2:** Tracer uptake (SUVmax) in tumor lesions

		FDG	FAPI	*P*_Wilcoxon_
SUVmax	Primary tumor (*n* = 15)			
	Median [Min, Max]	13.4 [5.68, 21.9]	14.8 [9.26, 26.6]	0.28
	Lymph node metastases (*n* = 23)			
	Median [Min, Max]	6.17 [1.73, 20.9]	9.47 [1.83, 24.9]	0.10
	Visceral metastases (*n* = 7)			
	Median [Min, Max]	5.57 [2.62, 11.1]	7.05 [1.80, 25.0]	0.46
	Bone metastases (*n* = 3)			
	Median [Min, Max]	2.59 [1.41, 2.75]	7.45 [4.00, 14.2]	0.25
SUVmean	Liver			
	Median [Min, Max]	2.58 [1.35, 3.88]	1.13 [0.400, 3.03]	< 0.001
	Bloodpool			
	Median [Min, Max]	2.12 [1.26, 2.88]	1.49 [0.970, 2.56]	< 0.001

**Table 3:** Tumor-to-background ratio (TBR)

		FDG (*n* = 15)	FAPI (*n* = 15)	*P*_Wilcoxon_
SUVmax tumor/SUVmean liver				
	Mean (SD)	3.41 (2.29)	9.99 (7.77)	< 0.001
	Median [Min, Max]	2.94 [0.461, 9.92]	8.70 [0.832, 37.7]	
SUVmax tumor/SUVmean bloodpool				
	Mean (SD)	4.28 (2.79)	7.48 (4.52)	< 0.001
	Median [Min, Max]	3.73 [0.576, 10.8]	7.10 [0.884, 16.5]	

TBR (SUVmax tumor/SUVmean liver and SUVmax tumor/SUVmean bloodpool) for FDG PET/CT and FAPI PET/CT

#### Lesion-Based Comparison of [^18^F]F-FDG PET/CT and [^68^ Ga]Ga-FAPI-46 PET/CT

A total of 49 lesions were recorded. Of these, 40 (82%) were FDG^+^ and 41 (84%) were positive by FAP^+^. There were five (10%) FAP^+^/FDG^−^ lesions and four (8%) FAP^−^/FDG^+^ lesions. This may be the result of tumor heterogeneity, since FAP displays CAFs and FDG tumor metabolism. Thus, [^68^ Ga]Ga-FAPI-46 PET/CT may not always be superior to [^68^F]F-FDG PET/CT as an imaging technique for all different tumor entities and differentiation grades. While knowledge of [^68^ Ga]Ga-FAPI-46 PET/CT remains incomplete, we recommend imaging with both tracers to obtain comprehensive diagnostic information. Disease stage identified by FAPI PET/CT was mostly concordant with FDG PET/CT. Compared to conventional imaging alone, five patients (33%) were upstaged following imaging by FAPI-PET/CT. All of these had visible lesions on FDG PET/CT as well.

**Fig. 2:**
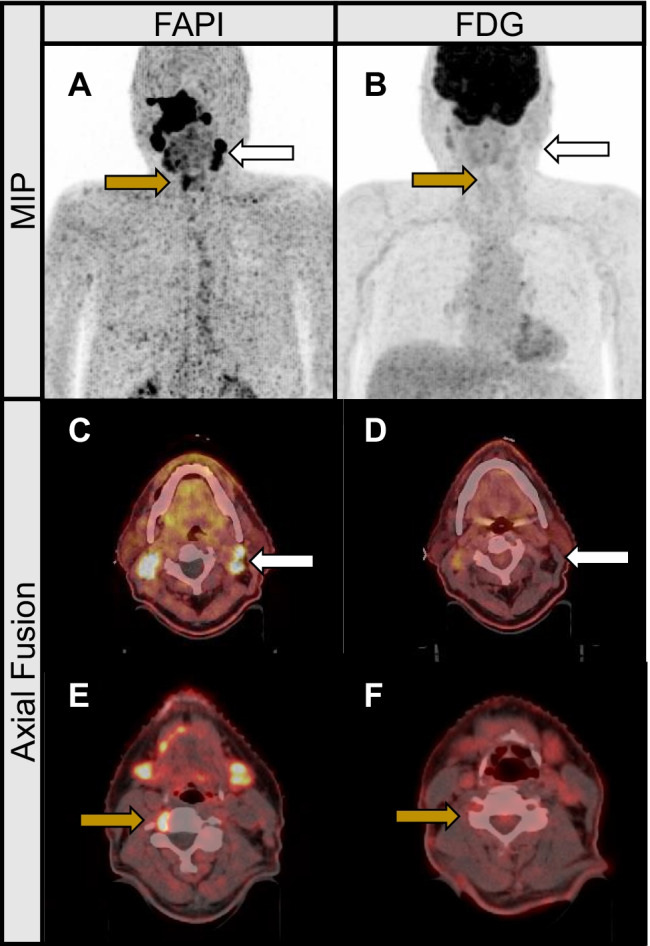
Cervical nodal metastasis with high FAPI uptake and low FDG uptake maximum intensity projections (**A**, **B**) and axial fusion images of a 73-year-old male with nasopharyngeal cancer prior to radiation therapy. The patient was upstaged with newly emerged nodal metastasis on both sides of the neck, noticed only in the FAP scans (**C**, **D**; white arrows). [.^68^ Ga]-FAPI PET/CT also revealed one cervical lymph node metastasis in close proximity to the cervical spinal cord (at C4 level) that would have been missed in the FDG-scans (**E**, **F**; yellow arrows).

In one case, a patient with nasopharyngeal cancer was upstaged with newly emerged nodal metastasis on both sides of the neck, noticed only in the FAP scans (see Fig. 2). [^68^ Ga]Ga-FAPI-46 PET/CT also revealed one cervical lymph node metastasis in close proximity to the cervical spinal cord (at C4 level) that would have been missed in the [^18^F]F-FDG scans. Standard cervical radiation would have left out this tumor manifestation with a subsequent high risk of recurrent or progressive disease in that region.

**Fig. 3:**
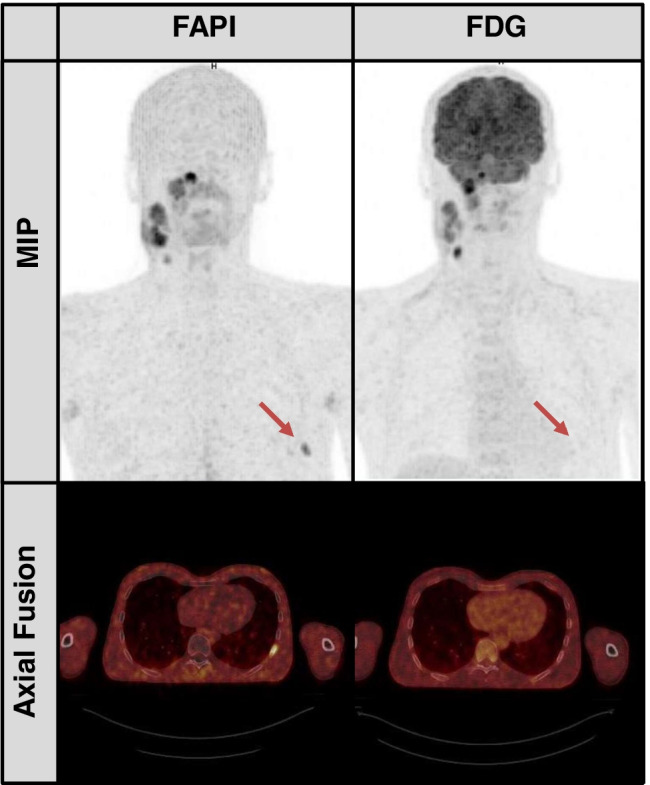
False-positive uptake in FAP-scans in a patient’s left rib. Maximum-intensity projections and axial fusion images of a 37-year-old male with nasopharyngeal cancer prior to radiation therapy. The patient had FAP uptake in the left 9th rib rated as benign (red arrow). Neither the FDG scan nor conventional imaging was suggestive of bone metastasis.

In two patients, we observed unspecific focal FAP-uptake in bone (in the clavicle and in a patient’s rib, see Fig. 3). As there was no correlation in the CT scan and no [^18^F]F-FDG uptake, we decided not to upstage these patients solely on the basis of the bone uptake in the FAP-scans.

#### Target Volumes (GTVs)

**Fig. 4:**
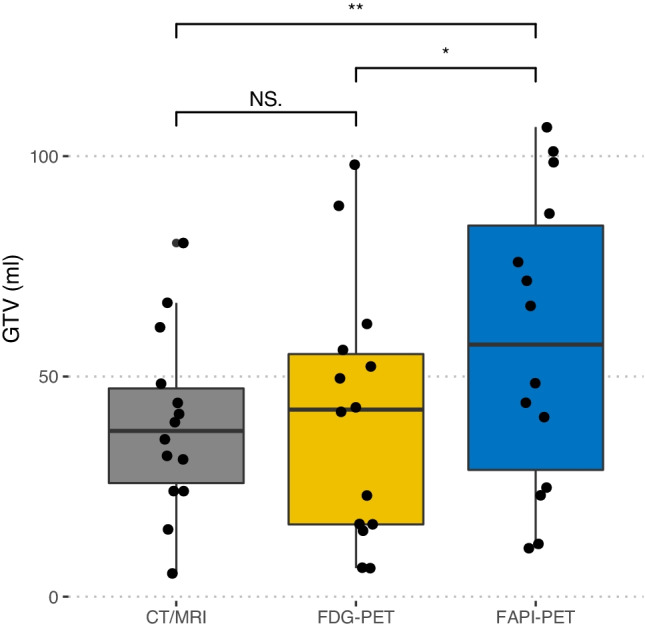
GTVs (ml) based on different imaging methods in 15 patients Grey box: GTV based on conventional imaging, mean volume: 39.2 ml (SD 20.3), yellow box: GTV based on FDG PET/CT, mean volume: 41.1 ml (SD 29.1) and blue box: GTV on the basis of FAPI PET/CT, mean volume: 57.9 ml (SD 33.4) (see Table 1). The statistical comparison was performed by a Wilcoxon paired rank test. NS not significant; *p < 0.05; **p < 0.01.

Target volumes were defined using VARIAN Eclipse software. Conventionally created GTVs (CONV-GTV, based on imaging information from MRI and CT) took up a median volume of 37.7 ml (range 5.3–80.3) (see Table 4; Fig. 4). Contoured GTVs based on imaging information from the FDG PET/CT (FDG-GTV; median 42.5 ml, range 6.5–98.1) were not significantly different to the CONV-GTVs (*p* = 0.95). The GTV created on the basis of [^68^ Ga]Ga-FAPI-46 PET/CT (FAPI-GTV; median 57.3 ml, range 11–107 ml) was significantly larger than CONV-GTV with a median increase of 9.2 ml (range − 12.0 to 58.1; *p* = 0.003) and FDG-GTV with a median increase of 7.2 ml (range − 12.0 to 66.6; *p* = 0.024). There was no case in which FAPI-GTV was smaller than the CONV-GTV. A few patients had [^68^ Ga]Ga-FAPI-46-uptake in their primary tumor regions, which would not have been covered by the CONV-GTV.

**Table 4:** Volumetric comparison of different gross tumor volumes (GTV)

	Overall (*n* = 15)
**GTV**_**conventional**_ (ml)	
Mean (SD)	39.2 (20.3)
Median [Min, Max]	37.7 [5.30, 80.3]
**GTV**_**FDG PET**_ (ml)	
Mean (SD)	41.1 (29.1)
Median [Min, Max]	42.5 [6.50, 98.1]
**GTV**_**FAPI PET**_ (ml)	
Mean (SD)	57.9 (33.4)
Median [Min, Max]	57.3 [11.0, 107]
**GTV**_**Final RT-plan**_ (ml)	
Mean (SD)	48.5 (36.7)
Median [Min, Max]	57.3 [5.3, 133.1]

GTVs were based on CE-CT and MRI (GTV_conventional_), based on FDG PET/CT (GTV_FDG PET_) and based on FAPI PET/CT (GTV_FAPI PET_). Final RT plan (GTV_Final RT-plan_) was made based upon all available imaging data, as well as clinical evaluation and interpretation of results

We observed a greater extent of FAP-uptake in primary tumor sites with higher tumor-to-background ratios and in some cases detected additional (contralateral) lymph nodes as well as further suspicious distant lesions.

#### Radiotherapy GTV and Outcome

FAPI-based GTVs were considered in ten patients (67%). Reasons for findings not being recorded as FAPI-positive lesions were FAPI-PET signal beyond morphological tumor volume considered as halo (*n* = 2), benign uptake surrounding tracheal stoma (*n* = 1), low FAPI-PET signal without MRI correlate (*n* = 1), and unclear pulmonary lesions confirmed as metastasis in a follow-up scan (*n* = 1). Final GTVs used for radiotherapy demonstrated very strong correlation with FAPI-GTV (rho = 0.72; *p* = 0.004), strong correlation with FDG-GTV (rho = 0.56; *p* = 0.0134), and strong but not statistically significant correlation with CONV-GTV (rho = 0.48; *p* = 0.0564, see Table 4). Outcome data is limited by heterogenous follow-up, as aftercare was not always performed at our institution. However, a minimum of 6 months of follow-up was available for all patients. After a median of 14.8 months of documented follow-up, five (33%) patients experienced progression. Of these, all patients had out-of-field progression at a median of 10.1 months. Only one patient had progression in a FAPI-positive region at baseline 4.5 months after the start of RT. This was a pulmonary lesion positive in both FDG- and FAPI-PET, retrospectively confirmed as lung metastasis. Three patients (20%) died during follow-up.

## Discussion

The present study demonstrates that [^68^ Ga]Ga-FAPI-46 PET/CT in HNC has promising features and may be at least equivalent to [^18^F]F-FDG PET/CT in terms of accuracy and useful not only for staging purposes but also and especially for radiotherapeutic treatment planning.

Our findings resulted in a higher median tumor GTV-volume in the FAP-scans while showing a favorable tumor-to-background ratio (TBR). This effect has been described by other groups, too [9]. HNCs tend to invade surrounding structures, sometimes impeding a clear differentiation between tumor and normal tissue in the imaging. Even when applying different thresholds for FAP-imaging (three-, five-, seven-, and tenfold increase of FAPI enhancement in the tumor as compared with normal tissue), other groups describe a good tumor-to-background ratio, especially in the cervical region, for [^68^ Ga]Ga-FAPI-46 PET/CT [14]. Like Giesel et al., we observe higher tumor-to-background ratios for FAPI compared to FDG, despite slightly lower absolute signal intensity of FAPI [15].

There is preliminary evidence that [^18^F]F-FDG PET/CT is able to detect HNC primary tumors as well as nodal and distant metastasis [16]. In their single-center exploratory study, Promteangtrong et al. find high accuracy for FAPI PET/CT in HNCs for primary staging and detection of recurrent tumors in twelve patients [17]. With respect to histopathological validation, both FDG- and FAPI-PET/CT exhibit high sensitivity (100%) and accuracy (94.4%). Future investigations confirming the high accuracy of FAP-tracers are still needed and should aim at histopathological correlation.

The role of CAFs in the tumor microenvironment is not entirely understood. There is some evidence that they can promote tumor progression and invasiveness as well as boosting antitumorigenic effects [18]. The finding in this study that FAPI-GTVs were larger than FDG-GTVs may reflect a peritumoral reaction of CAFs. For final radiotherapy contouring, we compared and merged the FDG-GTVs, the FAP-GTVs, and the imaging information obtained from CE-CT and MRI. In cases where the FAP-GTV suggested a greater volume, especially in the area of the tumor margin or in the adjacent surrounding tissue, we expanded the GTV for the final radiotherapy plan based on visual interpretation of findings and clinical expertise.

Although long-term survival rates of patients with HNCs increased, the recurrence rate of tumors treated with radiotherapy remained high (up to 50%) [19]. Insufficient radiation dose and growing radiation resistance of tumor cells were given as major reasons for tumor recurrence after radiotherapy. The effect of radiation on CAFs alters their tumor-promoting capability but unfortunately, treated CAFs show both enhancing and diminishing protumorigenic potential [19].

In our study, we found a similar uptake of [^68^ Ga]Ga-FAPI-46 in tumor lesions to that previously described by Syed et al. [14] However, the FAPI-based GTVs described by the Heidelberg group were greater when obtained by their FAPI × 3 method but smaller for all other GTVs. There are several possible reasons for this, one being that the tumor burden differed in the two cohorts. Importantly, we have chosen to determine the GTVs based on clinical contouring instead of purely on threshold, as we usually do with the imaging methods available.

At present, special caution is required when upstaging a patient based on [^68^ Ga]Ga-FAPI-46-imaging alone since, as with [^18^F]F-FDG-imaging, there are known to be various benign causes of uptake [20]. Clinical studies comparing imaging with FDG versus FAPI PET/CT for various types of cancer are emerging and hint at a superior diagnostic efficacy of FAPI in diagnosing primary and metastatic lesions in patients [21]. However, such results should be regarded as preliminary, as high-level evidence is still lacking.

This study benefits from the fact that immobilization devices were used for all imaging data, and our cohort of patients had had no prior treatment which could have distorted imaging quality. Performing the FDG-PET/CT and the FAPI-scans in the exact same arrangement as the one in which the patient would be positioned for the actual radiotherapy allowed a more precise comparison between the two scans and improved therapy planning. Furthermore, we provide comprehensive analysis on volume delineation for radiotherapy planning based on this novel tracer.

However, this study comes with limitations stemming primarily from the retrospective design and small sample size. Consequently, further studies are needed to determine whether radiotherapy adapted to FAPI-PET/CT can be translated into better local control and a sparing of organs at risk. Finally, superior oncologic outcomes need to be evaluated in prospective trials with sufficient cohort size. Further analysis of tumor relapse (in-field vs. margin vs. out-of-field-recurrence) would also be of great interest.

The incidence of HNC is growing while the population affected is getting younger [22], driving the need for more accurate diagnosing and treatment options not only prior to curative treatment but also in the case of tumor recurrence.

## Conclusion

Compared to [^18^F]F-FDG PET/CT and conventional imaging for staging and treatment planning in HNC, we found greater GTVs derived from [^68^ Ga]Ga-FAPI-46 PET/CT. In comparison to conventional imaging, both PET modalities revealed additional lesions in about one third of patients. Our results call for trials determining the clinical benefit of [^68^ Ga]Ga-FAPI-46 PET/CT-adapted dose mapping as well as the general diagnostic value of [^68^ Ga]Ga-FAPI-46 PET/CT in HNCs.

## Abbreviations

FAP: Fibroblast activation protein
[^68^ Ga]-FAPI: [^68^ Ga]-fibroblast activation protein inhibitor
PET: Positron emission tomography
CT: Computer tomography
[^18^F]-FDG: [^18^F]-fluorodeoxy-d-glucose
HNC: Head and neck cancer
CAFs: Cancer-associated fibroblasts
CE-CT: Contrast-enhanced computer tomography
GTV: Gross tumor volume
MRI: Magnetic resonance imaging
RT: Radiotherapy
SCC: Squamous cell carcinoma
TPS: Treatment planning system
CTV: Clinical target volume
PTV: Planning target volume
SUV: Standardized uptake value
EORTC: European Organisation for Research and Treatment of Cancer
IMRT: Intensity modulated radiotherapy
